# Hypertensive Disorders during Pregnancy (HDP), Maternal Characteristics, and Birth Outcomes among Japanese Women: A Hokkaido Study

**DOI:** 10.3390/ijerph18073342

**Published:** 2021-03-24

**Authors:** Kritika Poudel, Sumitaka Kobayashi, Chihiro Miyashita, Atsuko Ikeda-Araki, Naomi Tamura, Yu Ait Bamai, Sachiko Itoh, Keiko Yamazaki, Hideyuki Masuda, Mariko Itoh, Kumiko Ito, Reiko Kishi

**Affiliations:** 1 Faculty of Health Sciences, Hokkaido University, Sapporo 060-0812, Japan; kpoudel@hs.hokudai.ac.jp; 2 Center for Environmental and Health Sciences, Hokkaido University, Sapporo 060-0812, Japan; sukobayashi@cehs.hokudai.ac.jp (S.K.); miyasita@med.hokudai.ac.jp (C.M.); AAraki@cehs.hokudai.ac.jp (A.I.-A.); ntamura@cehs.hokudai.ac.jp (N.T.); u-aitbamai@med.hokudai.ac.jp (Y.A.B.); vzbghjn@den.hokudai.ac.jp (S.I.); kyamazaki@cehs.hokudai.ac.jp (K.Y.); hmasuda@cehs.hokudai.ac.jp (H.M.); mitoh@cehs.hokudai.ac.jp (M.I.); kumiko231627@gmail.com (K.I.); 3 Faculty of Health Sciences, Hokkaido University of Science, Sapporo 060-0812, Japan

**Keywords:** hypertensive disorders during pregnancy, mothers, birth outcomes, cohort study

## Abstract

Hypertension during pregnancy causes a greater risk of adverse birth outcomes worldwide; however, formal evidence of hypertensive disorders during pregnancy (HDP) in Japan is limited. We aimed to understand the association between maternal characteristics, HDP, and birth outcomes. In total, 18,833 mother-infant pairs were enrolled in the Hokkaido study on environment and children’s health, Japan, from 2002 to 2013. Medical records were used to identify hypertensive disorders and birth outcomes, namely, small for gestational age (SGA), SGA at full term (term-SGA), preterm birth (PTB), and low birth weight (LBW). The prevalence of HDP was 1.9%. Similarly, the prevalence of SGA, term-SGA, PTB, and LBW were 7.1%, 6.3%, 7.4%, and 10.3%, respectively. The mothers with HDP had increased odds of giving birth to babies with SGA (2.13; 95% Confidence Interval (CI): 1.57, 2.88), PTB (3.48; 95%CI: 2.68, 4.50), LBW (3.57; 95%CI: 2.83, 4.51) than normotensive pregnancy. Elderly pregnancy, low and high body mass index, active and passive smoking exposure, and alcohol consumption were risk factors for different birth outcomes. Therefore, it is crucial for women of reproductive age and their families to be made aware of these risk factors through physician visits, health education, and various community-based health interventions.

## 1. Introduction

Pregnancy-induced hypertension (PIH) is defined as hypertension (blood pressure ≥ 140/90 mmHg) with or without proteinuria (≥300 mg/24 h) emerging after 20 weeks of gestation. Furthermore, PIH is defined as a new onset proteinuria (≥300 mg/24 h) in hypertensive women exhibiting no proteinuria before 20 weeks of gestation [1]. In 2004, Japan revised the term “Toxemia of Pregnancy” to “Pregnancy Induced Hypertension,” which was further revised in 2017, to “Hypertensive disorders of Pregnancy,” shortly named HDP, which is consistent with the international classification [2]. HDP has been classified into four types as follows: preeclampsia, gestational hypertension, superimposed preeclampsia, and chronic hypertension, excluding eclampsia in the previous disease type classification [3]. HDP occurs in 5% of women and around 10% of primiparous women out of all pregnancies.

Maternal age, primiparity, Body Mass Index (BMI), multiple pregnancies, previous history of HDP, gestational diabetes mellitus, preexisting hypertension, preexisting type 2 diabetes mellitus, preexisting urinary tract infection, family history of hypertension, type 1 and type 2 diabetes, anti-phospholipid syndrome and systemic lupus erythematosus are non-modifiable risk factors of HDP [4,5]. HDP can trigger various maternal complications, including liver and kidney failure, cardiovascular diseases, placental abruption, disseminated intravascular coagulation, and hemolysis elevated liver enzyme low platelet count (HELLP) syndrome. These complications can cause placental dysfunction leading to fetal distress, intrauterine growth retardation, preterm birth (PTB), stillbirth, and neonatal asphyxia [6]. Mothers with hypertension during pregnancy have a greater risk of developing adverse pregnancy outcomes than normal pregnant women [7]. Studies conducted in United States, Italy, Canada, Haiti, Malaysia, and Nepal have shown that women with HDP are at double risk of PTB, at three-to four-fold risk of delivering small-for-gestational age (SGA) babies, and their neonates are at a higher risk of being admitted to neonatal intensive care units [8,9,10,11,12,13]. PTB has a higher perinatal mortality rate than term birth, whereas SGA has a slow postnatal growth and developmental delay [14].

Europe and the US have seen an increase in the ratio of overweight and obese women; however, Japan has seen a dramatic increase in the ratio of underweight women, especially between women in their 20s and 30s from the desire to lose weight [15,16,17]. Underweight pregnant women are more likely to have preterm delivery and giving birth to low birth weight (LBW) and SGA babies. The recent trend in Japan shows a declining mean birth weight from 3200 g to 3000 g (3050 g for boys; 2960 g for girls) within 43 years (1975 to 2018) [18,19]. Similarly, Japan’s maternal age at childbirth is increasing rapidly, with the mean age at first birth at 30.7 years in 2018, which was 25.7 years in 1975 [20,21,22]. A gradual trend towards delayed motherhood is seen in several countries with low fertility, such as Italy, Spain, China, Latin America, and North Africa [23].

Although the different causes for the increasing trends of adverse birth outcomes such as SGA, PTB, and LBW internationally and in Japan are unclear, studies have suggested that pregnancy at an older age, obesity, smoking during pregnancy, alcohol consumption, and HDP have been identified as risks [24]. To date, there are no reports to determine the proportion of HDPs in Japan. Therefore, this study aimed to determine the following (i) the risk factors of HDP among mothers participating in a Japanese prospective birth cohort, and (ii) the association of HDP and maternal characteristics with birth outcomes such as SGA, term-SGA, PTBs, and LBW. We hypothesized that the risk factors for HDP in pregnant Japanese women are similar to those in pregnant European and American women, and HDP increases the risk of SGA, term-SGA, PTB, and LBW.

## 2. Materials and Methods

### 2.1. Participants

**Figure 1:**
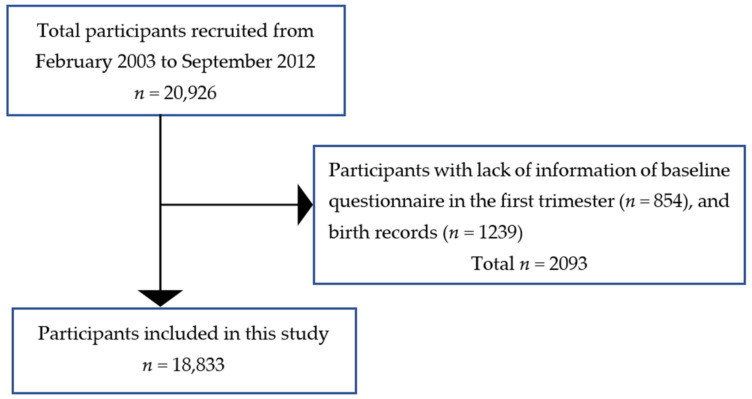
Flowchart of participants included in the statistical analyses.

The Hokkaido Study on Environment and Children’s Health is a prospective birth cohort that began in 2002. Further information about the aim of this study has been described previously [25,26,27]. From February 2003 to March 2012, the Hokkaido cohort included Japanese women who were recruited during early pregnancy (13 weeks of gestation) and who visited the maternity unit in one of the 37 hospitals and clinics in the Hokkaido Prefecture, Japan. These 37 health services cover the entire Hokkaido area. The cohort consisted of 20,926 pregnant women. As a current study focused on the birth outcomes, we included all mothers who completed the baseline questionnaire in the first trimester and with a medical record of their pregnancy. Participants who lacked this information were excluded from this study (*n* = 2093) (Figure 1). In total, we included the data obtained from 18,833 participants in the statistical analyses and assessed the associations between HDP and SGA, term-SGA, PTB, and LBW.

### 2.2. Questionnaire and Medical Records

After enrolling in the study, the participants completed a self-administered questionnaire, which consisted of information on parental characteristics. Questions on maternal age, height, weight, education, occupation, medical history, maternal smoking, and alcohol consumption during and before the first trimester, paternal smoking history, and use of any assisted reproductive technologies were asked. The medical record consisted of information about gestational days at delivery, live-birth, single or multiple births, sex, and birth weight of the infant. The medical history of HDP was obtained from medical records. We used Japanese standard definition of HDP and subclassified the symptoms based on severity. The mild HDP (h) refers to blood pressure ≥140/90 mmHg but <160/110 mmHg after 20 weeks of gestation, and proteinuria (*p*) ≥300 mg/24 h without exceeding 2.0 g/24 h or 3 + dipstick. The severe HDP (H) refers to blood pressure ≥160/110 mmHg and proteinuria (P) exceeding 2.0 g/24 h or 3 + dipstick [1].

### 2.3. Cotinine Level Measurements

Blood samples collected from participants during the third trimester of gestation were frozen at −80 °C. Cotinine measurement was carried out using the highly sensitive enzyme-linked immunosorbent assay (ELISA) technique. The limit of detection was 0.12 ng/mL. Non-detectable cotinine concentrations were assigned a half value of the detection limit (0.06 ng/mL) before the statistical analysis. The detailed experimental procedure has been discussed in our previous study [28,29]. We used the cotinine cut-off amounts based on our previous study to differentiate between passive and active smokers [28,29]. Based on this cotinine value, non-passive smokers (≤0.21 ng/mL), passive smokers (>0.21–≤11.48 ng/mL) and active smokers (>11.48 ng/mL) were defined.

### 2.4. Birth Outcomes

Our study discussed four birth outcomes as follows: SGA, term-SGA, PTB, and LBW. SGA was defined as newborns smaller in size than expected for their gestational age, expressed as a weight below the 10th percentile for the gestational age according to the condition of parity and the sex of the infant [30]. Term-SGA was described as a birth weight lower than the 10th percentile of the normative reference birth weight born >37 weeks of gestation (at full-term). PTB was defined as live birth at <37 weeks of gestation [31]. LBW was defined as a birth weight <2500 g. BMI was calculated from the pre-pregnancy weight. Asians are smaller than Caucasian people, hence, we used the database for birth weight published by the Japan Pediatric Society as a reference to calculate SGA and term-SGA [32].

### 2.5. Statistical Analysis

A descriptive analysis was performed to present the demographic and socio-personal information of the mothers. Binary logistic regressions were performed to evaluate association between maternal characteristics and HDP, maternal characteristics and birth outcomes, and HDP and birth outcomes with adjustment for the following covariates: maternal age, parity, smoking during the first trimester, and alcohol consumption during the first trimester. The confidence interval (CI) was set at 95%, with a level of significance at 0.05. All statistical analyses were conducted using IBM SPSS Statistics for Windows software version 22.0 (IBM Corp., Armonk, NY, USA).

## 3. Results

### 3.1. Characteristics of Infants and Mothers

Out of 18,833 mothers, 363 mothers developed HDP. There was a significant difference between the birth weight of infants, gestational age, and type of pregnancy among mothers with HDP compared to mothers without HDP. Similarly, there was a significant difference in age, BMI, parity, and fertility treatment for this pregnancy among mothers with HDP as compared to mothers without HDP. The characteristics of infants and mothers are presented in Table 1. There was no significant difference between mothers with and without HDP for types of delivery, education level, annual household income, drinking habit, smoking habit, occupation, exposure to chemicals, drip infusion, and plasma cotinine levels. In addition, no significant difference was observed in the characteristics in the education of partner, medical illness, occupation, exposure to chemicals, smoking, and drinking habits.

### 3.2. Maternal Characteristics and HDP

Table 2 presents the association between maternal characteristics and HDP. The crude model showed that the risk of HDP was significantly higher among older mothers, high BMI, multiple pregnancy, those who received fertility treatment, and those who underwent in vitro fertilization (IVF) for this pregnancy and the decreased risk for multiparous. The crude model showed no association between HDP and plasma cotinine level. The adjusted model showed that the risk of HDP was higher among older mothers, high BMI, twin pregnancy, those who received in vitro fertilization for this pregnancy and active smokers. There was no association between HDP and parity in the adjusted model. No significant association was observed between HDP and maternal characteristics such as education level, annual household income, medical illness, exposure to chemicals, and occupation.

### 3.3. Maternal Characteristics and Birth Outcomes

Table 3 presents the association between maternal characteristics and SGA and term-SGA. The crude model showed that SGA was associated with the increased risk for mothers with a low BMI, twin pregnancy, smoking habit, smoking habit before and during pregnancy, drinking habit before pregnancy, those who received fertility treatment for this pregnancy, and active smokers as per plasma cotinine levels during the third trimester, and had the decreased risk for multiparity. After adjustment, SGA was associated with older age, low BMI, twin pregnancy, smoking habit during pregnancy, drinking habit before pregnancy, and active smokers as per plasma cotinine level during the third trimester.

The crude model showed that term-SGA was associated with mothers with low BMI, smoking habits before and during pregnancy, drinking habits before pregnancy, and active smokers as per plasma cotinine levels during the third trimester. The adjustment model did not show any association of term-SGA with the smoking habit, smoking habit before pregnancy, and drinking habits compared to the crude model.

Table 4 presents the association between maternal characteristics and PTB and LBW. The crude model showed that PTB was associated with older age, high BMI, twin pregnancy, smoking habit before pregnancy, those who received fertility treatment for this pregnancy, and those who underwent IVF. The adjusted model showed similar findings for the association of maternal characteristics and PTB.

LBW was significantly associated with older age, low BMI, twin pregnancy, smoking habit during pregnancy, drinking habit before pregnancy, those who underwent fertility treatment for this pregnancy, those who underwent IVF, and active smokers as per plasma cotinine levels during the third trimester.

### 3.4. Association between HDP and Birth Outcomes

Table 5 presents the association between HDP and birth outcomes. The crude and adjusted model showed that HDP was found to be significantly associated with SGA, PTB, and LBW.

## 4. Discussion

This study has tried to understand the risk factors of HDP, and the association between maternal characteristics and HDP on birth outcomes such as SGA, term-SGA, PTBs, and LBW. HDP was found to be associated with SGA (2.13 OR), PTB (3.48 OR), and LBW (3.57 OR). Our findings are similar to previous studies, which have suggested a higher risk of perinatal outcomes among women with HDP [33,34]. There was no association between term-SGA and HDP in our study. PTBs have become a significant public health issue worldwide and constitute a significant cause of infant mortality and low infant development [35]. More than 60% of PTB occurs in Africa and South Asia; furthermore, on average, 12% of babies are born too early in lower-income countries, compared with 9% in higher-income countries [36]. Studies have indicated that increasing maternal age at delivery, and primiparity are a few of the few reasons that have been found to increase PTB rates [35,37]. Our study also showed an increased risk of PTB among women who were pregnant at an older age, had a high BMI, had a habit of smoking before pregnancy, and a mother who opted for IVF. Other studies have identified adolescent birth rate, short stature among women of childbearing age, underweight mothers, obesity, multifetal pregnancy, pregnancies spaced too closely, and antepartum hemorrhage as the possible causes of PTB in lower-income countries [38,39].

HDP and its subtypes are one of the major risk factors for the development of cerebral, renal, cardiovascular disease (CVD) in the mothers in later phase of life [5]. Similarly, studies have shown that babies born to mothers with HDP are at greater risk of noncommunicable diseases, women with preeclampsia are at high risk of developing stroke, hypertension, diabetes mellitus, end renal stage disease and CVD when they become adults [40]. The HDP prevalence was 1.9% in our study, which was less than the Japan Environment and Children’s Study (3.1%), a nationwide birth cohort study [41]. The difference might be due to variation in the study group and Japanese population of pregnant women. The prevalence of HDP in our study was lower than that reported for Chinese (5.2%), African Americans (6.4%), and Brazilian (7.5%) studies [6,42,43]. The differences might occur due to variation in age distribution, socio-economic backgrounds, ethnicity, and the enhancement of the Japanese health care delivery system, which prioritizes health checkups during pregnancy.

The prevalence of LBW deliveries in high-income countries is lower (7%) as compared to low-income countries (12–25%) [44]. Additionally, in lower-income countries, about one in five infants are born SGA, and one in four neonatal deaths occur in SGA babies [45]. From 1985 to 2013, infants’ mean birth weight in Japan reduced to 3000 g (from 3120 g) and LBW babies increased from 6.3% to 9.6% [32]. In Japan, the number of underweight women, mainly in their 20s and 30s, has significantly increased. A survey conducted by the Ministry of Health, Labor and Welfare showed that about 25% of Japanese women of reproductive age are underweight (BMI < 18.5 kg/m^2^) and are at risk of delivering LBW infants and SGA infants [21,46,47]. Our study showed that pre-pregnancy BMI (<18.5 kg/m^2^) showed a strong association with SGA, term SGA, PTB, and LBW. Low weight gain before and during pregnancy causes inadequate nutrient supply to the fetus and can induce poor head circumference growth in newborns [48]. This could result in fetal malnutrition, leading to various organ developmental failures. While Japan’s population growth rate continues to decrease, the tendency to maintain low BMI among mothers could become a significant risk for neonatal mortality. Hence, effective health education about nutritious diet and healthy weight gain from an early stage in school life, receiving proper counseling from medical doctors, gynecologists, and nurses regarding the relationship between BMI and LBW can prevent LBW infant deliveries in the future.

An Italian population-based study revealed that low maternal education was a risk factor for PTB, LBW, and SGA [49]. No such association was found in our study. This might be because the education level of mothers in our study was higher than that of Italy. Several studies have suggested primiparity as a risk factor for PTB [50]; however, primiparity was found to be associated with SGA, term-SGA, and LBW in our study. Our findings are consistent with a previous Japanese study, which indicated that the risk of LBW is lower among multiparous women [21]. Our findings are consistent with other studies that showed an association between birth outcomes and maternal characteristics, such as LBW babies and PTB with maternal age [43,51] and LBW and PTB with maternal BMI [30,52]. This suggests that women who want to conceive their first child at a later age and who have a low or high BMI should be counseled by their family health practitioners and be continuously monitored to reduce the occurrence of birth complications.

Our study showed a strong association between twin and multiple pregnancies and HDP. Previous studies have shown an increased risk for gestational hypertension, preeclampsia, and eclampsia in twin pregnancies compared to singleton pregnancies in primiparous and multiparous women [53]. Similarly, twin pregnancy was associated with SGA, PTB, and LBW in this study. Studies suggest that PTB is more common among women with twin pregnancies than singleton pregnancies, and HDP is more common in multiple than singleton pregnancies [54].

This study showed a strong association of fertility treatment, mainly IVF, with PTB and LBW. Several studies have shown the risk of preterm delivery and LBW babies among women conceiving after fertility treatment [55,56]. The proportion of twin births was higher among women receiving fertility treatment in our study (14.8% vs. 1.3%). IVF pregnancies are precious to infertile couples, leading to labor induction, and elective cesarean section, causing an iatrogenic increase in preterm delivery and an increased risk of preterm delivery among infertile couples. A study has shown that IVF increased the risk of LBW by 3.78, similar to our study (OR: 3.62) [55]. However, the OR reduced to 3.27 when adjusted for age, parity, BMI, smoking, and alcohol consumption. As these factors affect pregnancy outcomes, they should be considered while studying the effect of fertilization on different birth outcomes. This finding is similar to our previous study that showed different risk factors for term-SGA, LBW, and PTBs in the Japanese setting; however, this study did not include mothers with HDP [31].

The smoking rate among females in Hokkaido is 16.1%, which is higher than the national average for women (9.5%) and highest among all prefectures in Japan [57]. The smoking rate among males in Hokkaido is 34.6%, which is higher than the national average smoking rate for males (31.1%) and is the fourth highest compared to other prefectures [58]. The increasing smoking habits among females and increased exposure to secondhand smokers during pregnancy can cause intrauterine growth retardation and decreased birth weight among infants and children. A Japanese study has shown the reduction of birth weight by 125–136 g among smoking mothers during pregnancy [59]. Our study showed that smoking habits before pregnancy were associated with SGA, term-SGA, and PTB, while smoking during pregnancy was associated with SGA, term-SGA, and LBW. Active smoking during the third trimester (plasma cotinine level >11.48 ng/mL) was significantly associated with SGA, term-SGA, and LBW. Smoking before and during pregnancy has a negative impact on newborn babies, either in terms of weight or gestational age. Smoking during the first and third trimester has a similar impact on neonates. Our previous study showed that passive pregnant smokers with cotinine levels ≥3.03 ng/mL had an almost equal risk of delivering SGA infants as active smokers with cotinine levels (>11.48 ng/mL) [29]. Few studies focusing on the effects of maternal smoking during pregnancy and HDP have shown that smoking reduces the risk of preeclampsia and HDP [60,61]. Our study showed a significant association between plasma cotinine levels during the third trimester and HDP when controlled for age, parity, BMI, smoking during pregnancy, and alcohol consumption during pregnancy. Our study showed an association between HDP and passive smokers (>0.21 to ≤11.48 ng/mL) and active smokers (>11.48 ng/mL). Hence, women of reproductive age must be aware of prenatal smoking exposure on their babies and the risk of abnormal birth outcomes. A Polish study suggested that the risk of PIH increased significantly among women who smoked in the first trimester and with a low BMI before pregnancy [62]. The increased risk of HDP should be monitored among passive and active smokers, in addition to adverse birth outcomes [63].

There was an association between SGA, term-SGA, and LBW babies among mothers who habitually drank alcohol before pregnancy. However, we did not find any association between alcohol consumption before pregnancy and during the first trimester and preterm delivery risk. This might have occurred because our alcohol consumption data were based on questionnaires, and not on direct observation or volume calculation. A Japanese study found that maternal alcohol consumption during pregnancy was significantly associated with an increased PTB risk but did not show any relationship with LBW and SGA [64,65]. A meta-analysis on the dose-response relationship has shown that heavy alcohol consumption increases the risks of SGA, PTB, and LBW [66]; however, further studies need to be conducted in an Asian context to understand the different doses of alcohol and their effect during pregnancy. It is highly recommended to avoid alcohol consumption during pregnancy to reduce the onset of HDP among women in Japan [32].

A Japanese national survey conducted over 55 years (1961–2016) has shown a steady decrease in systolic blood pressure levels among all age groups of men and women, but not in the diastolic blood pressure levels requiring different population-based strategies to manage and prevent hypertension [67].

The strength of this study is its prospective birth cohort study design comprising 18,833 mother-infant pairs. In this study, we calculated the HDP from the medical records, which is a highly reliable report. We measured cotinine levels during the eighth month of pregnancy, as maternal smoking during the third trimester was related to birth size reduction. However, this study has some limitations. The participants who had HDP were relatively small (1.9% of the total participants), which might have led to an underestimation of the results. The study participants were pregnant women who had visited hospitals or clinics within the Hokkaido prefecture only. The pre-pregnancy weight, maternal smoking and alcohol consumption habit were self-reported by the respondents, which might have caused biasness. As we did not have participants with chronic hypertension, they were not included in the analysis. In addition, the reported risk of gestational diabetes mellitus, type 2 diabetes mellitus, family history of hypertension, and preexisting urinary tract infection were not assessed in this study.

## 5. Conclusions

Our study showed that mothers with HDP had increased odds of giving birth to babies with SGA, PTB, and LBW than mothers with normotensive pregnancy. Therefore, women of reproductive age need to be made aware of different risk factors through physician visits, health education, and various community interventions related to public health. Risk assessment before pregnancy, management of modifiable risk factors, and medical history monitoring should be implemented as per national guidelines to reduce further complications among pregnant mothers with HDP. It is crucial to remember that these risk factors can cause maternal and neonatal complications in mothers with HDP. Our study recommends future studies on Japanese mothers with HDP to demonstrate an association between characteristics and birth outcomes, considering the mediating effects of different confounding variables.

## Figures and Tables

**Table 1:** Characteristics of infants and mothers.

Characteristics	All (*n* = 18,833)	No HDP (*n* = 18,470)	With HDP (*n* = 363)	*p*-Value
**Mothers**				
Maternal age (years)				
≤24	2232	2184 (11.8)	36 (9.9)	<0.001
25–34	12,440	12,229 (66.2)	211 (58.1)	
≥35	3878	3765 (20.4)	113 (31.1)	
Missing	283	292 (1.6)	3 (0.8)	
BMI (kg/m^2^)				
<18.5	3177	3139 (17.0)	38 (10.5)	<0.001
18.5–24.9	13,214	12,995 (70.4)	219 (60.3)	
25.0–29.9	1514	1453 (7.9)	61 (16.8)	
≥30.0	464	428 (2.3)	36 (9.9)	
Missing	464	455 (2.5)	9 (2.5)	
Parity				
Primiparous	5951	5790 (31.3)	160 (44.1)	<0.001
Multiparous	12,646	12,451 (67.4)	196 (54.0)	
Missing	236	229 (1.2)	7 (1.9)	
Fertility treatment for this pregnancy				
Artificial insemination	238	232 (1.3)	6 (1.7)	<0.001
In vitro fertilization	284	266 (1.4)	18 (5.0)	
Ovulation inducer	565	552 (3.0)	13 (3.6)	
HDP (medical record) (multiple choice allowed)				
h (Hypertension 140–160/90–110 mmHg)	125		125 (34.4)	
H (Hypertension >160/110 mmHg)	93		93 (25.6)	
*p* (Proteinuria 0.3 g/24 h)	139		139 (38.3)	
P (Proteinuria >2 g/24 h)	67		67 (18.4)	
s (Superimposed type)	15		15 (4.1)	
Unspecified Hypertension	36		36 (9.9)	
Annual household income (million JPY)				
>3	3656	3589 (19.4)	67 (18.5)	0.653
3 to <5	7122	6988 (37.8)	134 (36.9)	
5 to <8	4072	3983 (21.6)	89 (24.5)	
>8	1199	1176 (6.4)	23 (6.3)	
Missing	2784	2734 (14.8)	50 (13.8)	
Smoking habit				
Yes	9963	9765 (52.9)	155 (42.7)	0.383
No	8556	8401 (45.5)	198 (54.5)	
Missing	314	304 (1.6)	10 (2.8)	
Drinking habit				
Yes	11,420	11,205 (60.7)	142 (39.1)	0.682
No	7219	7077 (38.3)	215 (59.2)	
Missing	194	188 (1.0)	6 (1.7)	
Plasma cotinine level (3rd trimester ng/mL)				
Non-passive smoker (≤0.21)	6183	6093 (33.0)	90 (24.8)	0.127
Passive smokers (>0.21–≤11.48)	7025	6897 (37.3)	128 (35.3)	
Active smokers (>11.48)	2291	2245 (12.2)	46 (12.7)	
Missing	3334	3235 (17.5)	99 (27.3)	
**Infants**				
Birth weight				
Equal or more than 2500 g	16,499	16,242 (87.9)	257 (70.8)	<0.001
Less than 2500 g	1945	1841 (10.0)	104 (28.7)	
Missing	389	387 (2.1)	2 (0.6)	
Small for gestational age (SGA)	1340	1290 (7.0)	50 (13.8)	<0.001
Term-SGA	1194	1169 (6.3)	25 (6.9)	0.666
Gestational age				
Preterm (<37 weeks)	1387	1310 (7.1)	77 (21.2)	<0.001
Full term (≥37 weeks)	17,152	16,867 (91.3)	285 (78.5)	
Missing	294	293 (1.6)	1 (0.3)	
Types of pregnancy				
Singleton	18,456	18,126 (98.1)	330 (90.9)	<0.001
Twin	368	337 (1.8)	31 (8.5)	
Multiple	9	7 (0.1)	2 (0.6)	

Chi-squared test; SGA, small for gestational age; weight Z-score, gestational age-specific-Z-score; cut-off for plasma cotinine level, 0.21 ng/mL (value differentiating non passive smokers from passive smokers among non-active smokers), 11.48 ng/mL (value differentiating non-active smokers from active smokers) [28].

**Table 2:** Association between maternal characteristics and hypertensive disorders during pregnancy (significant only).

Maternal Characteristics	*n*_all_ (18,833)	*n*_case_ (363)	Hypertensive Disorders during Pregnancy	
			Crude OR	aOR
Maternal age (years)				
≤24	2220	36 (9.9)	Reference	
25–34	12,440	211 (58.1)	1.05 (0.73, 1.49)	1.08 (0.70, 1.67)
≥35	3878	113 (31.1)	1.82 (1.25, 2.66) **	1.68 (1.04, 2.72) *
Missing	295	3 (0.8)		
BMI (kg/m^2^)				
<18.5	3177	38 (10.5)	0.72 (0.51, 1.02) ^ꝉ^	0.61 (0.38, 0.96) *
18.5–24.9	13,214	219 (60.3)	Reference	
25.0–29.9	1514	61 (16.8)	2.49 (1.87, 3.33) ***	2.55 (1.76, 3.69) ***
≥30.0	464	36 (9.9)	4.99 (3.46, 7.19) ***	6.90 (4.53, 10.48) ***
Missing	464	9 (2.5)		
Parity				
Primiparous	5950	160 (44.1)	Reference	
Multiparous	12,647	196 (54.0)	0.57 (0.46, 0.70) ***	0.49 (0.37, 0.64)
Missing	236	7 (1.9)		
Types of pregnancy				
Singleton	18,456	330 (90.9)	Reference	
Twin	368	31 (8.5)	5.05 (3.44, 7.41) ***	3.54 (2.05, 6.11) ***
Multiple	9	2 (0.6)	15.69 (3.25, 75.83) **	6.08 (0.73, 50.51)
Fertility treatment for this pregnancy				
No	17,764	325 (89.5)	Reference	
Yes	924	34 (9.4)	2.05 (1.43, 2.94) ***	1.15 (0.69, 1.92)
Missing	145	4 (1.1)		
Artificial insemination				
No	18,595	357 (98.3)	Reference	
Yes	238	6 (1.7)	1.32 (0.58, 2.99)	1.02 (0.36, 2.83)
In vitro fertilization				
No	18,549	345(95.0)	Reference	
Yes	284	18 (5.0)	3.57 (2.19, 5.82) ***	2.22 (1.11, 4.42) *
Plasma cotinine level (3rd trimester ng/mL)				
Non-passive smokers (≤0.21)	6183	90 (24.8)	Reference	
Passive smokers (>0.21–≤11.48)	7025	128 (36.2)	1.26 (0.96, 1.65)	1.44 (1.01, 2.06) *
Active smokers (>11.48)	2291	46 (12.7)	1.39 (0.97, 1.98) ^ꝉ^	2.27 (1.29, 4.00) **
Missing	3334	99 (27.3)		

Note: ^ꝉ^ *p* < 0.1, * *p* < 0.05, ** *p* < 0.01, *** *p* < 0.001. Confidence interval, 95%; Crude OR, crude odds ratio; aOR, adjusted odds ratio for age, parity, smoking during the first trimester, drinking alcohol during the first trimester. Plasma cotinine level cut-off value, 0.21 ng/mL [28].

**Table 3:** Maternal characteristics and birth outcomes, small for gestational age (SGA) and Term-SGA.

Characteristics	*n*_total_	SGA (*n* = 1340)			Term-SGA (*n* = 1194)		
		*n*_case_	Crude OR	aOR	*n*_case_	Crude OR	aOR
Maternal age (years)							
24	2220	150	Reference		134	Reference	
25–34	12,440	889	1.06 (0.89, 1.27)	1.20 (0.96, 1.51)	803	1.07 (0.89, 1.30)	1.19 (0.94, 1.51) ^ꝉ^
≥35	3878	301	1.16 (0.95, 1.42)	1.47 (1.13, 1.92) **	257	1.10 (0.89, 1.37)	1.36 (1.03, 1.79) *
Missing	295						
BMI (kg/m^2^)							
<18.5	3177	345	1.76 (1.54, 2.01) ***	1.95 (1.66, 2.30) ***	311	1.78 (1.55, 2.04) ***	1.98 (1.67, 2.35) ***
18.5–24.9	13,214	855	Reference		761	Reference	
25.0–29.9	1514	80	0.81 (0.64, 1.02)	0.72 (0.53, 0.98) *	66	0.74 (0.58, 0.96) *	0.69 (0.49, 0.96) *
≥30.0	464	23	0.75 (0.49, 1.15)	0.47 (0.25, 0.89) *	19	0.70 (0.44, 1.11)	0.48 (0.25, 0.94) *
Missing	464	37			37		
Parity							
Primiparous	5950	462	Reference		412	Reference	
Multiparous	12,647	848	0.85 (0.76, 0.96) **	0.81 (0.69, 0.94) **	759	0.86 (0.76, 0.97) *	0.82 (0.70, 0.96) *
Missing	236	30			23		
Types of pregnancy							
Singleton	18,456	1263	Reference		1168	Reference	
Twin	368	76	3.54 (2.73, 4.59) ***	3.57 (2.51, 5.08) ***	26	1.12 (0.75, 1.68)	1.14 (0.66, 1.97)
Multiple	9	1	1.70 (0.21, 13.62)	2.13 (0.26, 17.40)	0		
Smoking habit							
No	8556	555	Reference		484	Reference	
Yes	9963	761	1.19 (1.06, 1.33) **	1.05 (0.89, 1.25)	688	1.24 (1.10, 1.39) **	1.12 (0.94, 1.34)
Missing	314	24			22		
Smoking habit before pregnancy							
No	11,388	766	Reference		669	Reference	
Yes	6835	521	1.14 (1.02, 1.28) *	1.01 (0.85, 1.21)	476	1.20 (1.06, 1.35) **	1.06 (0.88, 1.27)
Missing	610	53			49		
Smoking habit during pregnancy							
No	13,532	868	Reference		771	Reference	
Yes	2155	243	1.85 (1.60, 2.15) ***	1.98 (1.66, 2.37) ***	223	1.91 (1.63, 2.23) ***	2.07 (1.72, 2.50) ***
Missing	3146	229			200		
Drinking habit							
No	7219	453	Reference		406	Reference	
Yes	11,420	868	1.23 (1.09, 1.38) **	1.15 (0.99, 1.33) ^ꝉ^	770	1.21 (1.07, 1.37) **	1.14 (0.98, 1.33) ^ꝉ^
Missing	194	19			18		
Drinking habit before pregnancy							
No	16,378	1099	Reference		973	Reference	
Yes	2178	215	1.52 (1.31, 1.77) ***	1.56 (1.26, 1.92) ***	197	1.57 (1.34, 1.85) ***	1.61 (1.29, 2.00) ***
Missing	277	26			24		
Fertility treatment for this pregnancy							
No	17,764	1244	Reference		1122	Reference	
Yes	924	84	1.33 (1.05, 1.67) *	1.32 (0.97, 1.80) ^ꝉ^	63	1.08 (0.83, 1.41)	1.03 (0.72, 1.48)
Missing	145	12			9		
In vitro fertilization							
No	18,549	1316	Reference		1179	Reference	
Yes	284	24	1.21 (0.79, 1.84)	1.25 (0.71, 2.19)	15	0.82 (0.49, 1.39)	0.58 (0.25, 1.32)
Plasma cotinine level (3rd trimester ng/mL)							
Non-passive	6183	374	Reference		348	Reference	
Passive	7025	471	1.12 (0.97, 1.28)	1.09 (0.91, 1.32)	426	1.08 (0.93, 1.25)	1.06 (0.88, 1.29)
Active	2291	241	1.83 (1.54, 2.16) ***	1.46 (1.07, 1.99) *	233	1.90 (1.60, 2.26) ***	1.57 (1.15, 2.16) **
Missing	3334						

^ꝉ^*p* < 0.1, * *p* < 0.05, ** *p* < 0.01, *** *p* < 0.001. Confidence interval, 95%; Crude OR, Crude Odds Ratio; aOR, adjusted odds ratio for age, parity, smoking during the first trimester, drinking alcohol during the first trimester.

**Table 4:** Maternal characteristics and birth outcomes.

Characteristics	*n*_total_	Preterm Birth (*n* = 1387)			Low Birth Weight (*n* = 1945)		
		*n*_case_	Crude OR	aOR	*n*_case_	Crude OR	aOR
Maternal age (years)							
≤24	2220	127	Reference		196	Reference	
25–34	12,440	878	1.25 (1.03, 1.52) *	1.31 (1.03, 1.67) *	1241	1.15 (0.98, 1.34) ^ꝉ^	1.28 (1.05, 1.56) *
≥35	3878	382	1.80 (1.46, 2.22) ***	1.92 (1.48, 2.50) ***	507	1.56 (1.31, 1.86) ***	1.89 (1.51, 2.36) ***
Missing	295				1		
BMI (kg/m^2^)							
<18.5	3177	259	1.16 (1.00, 1.34) *	1.26 (1.06, 1.50) **	444	1.51 (1.34, 1.70) ***	1.59 (1.37, 1.83) ***
18.5–24.9	13,214	939	Reference		1289	Reference	
25.0–29.9	1514	115	1.08 (0.88, 1.32)	0.96 (0.74, 1.25)	119	0.79 (0.65, 0.96) *	0.62 (0.48, 0.81) ***
≥30.0	464	51	1.62 (1.20, 2.19) **	1.80 (1.27, 2.55) **	55	1.25 (0.94, 1.67)	1.18 (0.83, 1.68)
Missing	464	23			38		
Parity							
Primiparous	5950	423	Reference		667	Reference	
Multiparous	12,647	943	1.04 (0.93, 1.18)	0.97 (0.84, 1.13)	1243	0.86 (0.77, 0.94) **	0.82 (0.72, 0.93) **
Missing	236	21			35		
Types of pregnancy							
Singleton	18,456	1106	Reference		1642	Reference	
Twin	368	272	44.63 (35.03, 56.86) ***	44.06 (32.20, 60.30) ***	294	42.02 (32.22, 54.80) ***	45.95 (32.58, 64.80) ***
Multiple	9	9			9		
Smoking habit							
No	8556	667	Reference		864	Reference	
Yes	9963	707	0.90 (0.81, 1.00) ^ꝉ^	0.87 (0.74, 1.02) ^ꝉ^	1048	1.04 (0.95, 1.15)	0.96 (0.84, 1.11)
Missing	314	13			33		
Smoking habit before pregnancy							
No	11,388	882	Reference		1156	Reference	
Yes	6835	466	0.87 (0.77, 0.97) *	0.83 (0.71, 0.99) *	720	1.04 (0.94, 1.15)	0.95 (0.82, 1.10)
Missing	610	39			69		
Smoking habit during pregnancy							
No	13,532	999	Reference		1326	Reference	
Yes	2155	164	1.03 (0.87, 1.22)	0.93 (0.75, 1.14)	308	1.53 (1.34, 1.75) ***	1.58 (1.35, 1.86) ***
Missing	3146	224			311		
Drinking habit							
No	7219	551	Reference		704	Reference	
Yes	11,420	828	0.95 (0.85, 1.06)	0.94 (0.82, 1.08)	1222	1.11 (1.01, 1.23) *	1.04 (0.92, 1.17)
Missing	194	8			19		
Drinking habit before pregnancy							
No	16,378	1214	Reference		1646	Reference	
Yes	2178	164	1.02 (0.86, 1.20)	0.93 (0.72, 1.18)	276	1.30 (1.13, 1.49) ***	1.18 (0.97, 1.43) ^ꝉ^
Missing	277	9			23		
Fertility treatment for this pregnancy							
No	17,764	1184	Reference		1707	Reference	
Yes	924	192	3.69 (3.11, 4.37) ***	3.57 (2.88, 4.44) ***	222	3.00 (2.55, 3.51) ***	2.90 (2.35, 3.56) ***
Missing	145	11			16		
In vitro fertilization							
No	18,549	1306	Reference		1865	Reference	
Yes	284	81	5.42 (4.16, 7.07) ***	5.63 (3.99, 7.93) ***	80	3.62 (2.78, 4.72) ***	3.27 (2.30, 4.67) ***
Plasma cotinine level (3rd trimester ng/mL)							
Non-passive	6183	291	Reference		435	Reference	
Passive	7025	308	0.93 (0.79, 1.09)	0.93 (0.76, 1.15)	551	1.12 (0.99, 1.28) ^ꝉ^	1.06 (0.89, 1.25)
Active	2291	97	0.89 (0.71, 1.13)	1.00 (0.68, 1.49)	263	1.71 (1.45, 2.01) ***	1.36 (1.02, 1.81) *
Missing	3334						

^ꝉ^*p* < 0.1, * *p* < 0.05, ** *p* < 0.01, *** *p* < 0.001; Confidence interval, 95%; Crude OR, crude odds ratio; aOR, adjusted odds ratio for age, parity, smoking during the first trimester, drinking alcohol during the first trimester.

**Table 5:** Association between hypertensive disorders during pregnancy and small for gestational age (SGA), term-SGA, preterm birth, and low birth weight.

Group	*n*_all_	*n*_case_	Crude OR	*p*	aOR	*p*
HDP	**SGA**					
No	18,470	1290	Reference		Reference	
Yes	363	50	2.13 (1.57, 2.88)	<0.001	2.14 (1.41, 3.26)	<0.001
HDP	**Term-SGA**					
No	18,470	1169	Reference		Reference	
Yes	363	25	1.09 (0.73, 1.65)	0.666	1.34 (0.79, 2.26)	0.272
HDP	**PTB**					
No	18,470	1310	Reference		Reference	
Yes	363	77	3.48 (2.68, 4.50)	<0.001	2.94 (2.09, 4.13)	<0.001
HDP	**LBW**					
No	18,470	1841	Reference		Reference	
Yes	363	104	3.57 (2.83, 4.51)	<0.001	4.10 (3.04, 5.54)	<0.001

Confidence interval, 95%; Crude OR, crude odds ratio; aOR, adjusted odds ratio for age, parity, smoking during the first trimester, drinking alcohol during the first trimester; SGA, small for gestational age; PTB, preterm birth; LBW, low birth weight.

## Data Availability

The data are not publicly available due to ethical restrictions and specific legal framework in Japan. All inquiries should be addressed to Reiko Kishi, investigator of the Hokkaido Study on Environment and Children’s Health, Center for Environmental and Health Sciences, Hokkaido University.
